# ERAD and protein import defects in a *sec61* mutant lacking ER-lumenal loop 7

**DOI:** 10.1186/1471-2121-14-56

**Published:** 2013-12-06

**Authors:** Thomas Tretter, Fábio P Pereira, Ozlem Ulucan, Volkhard Helms, Susanne Allan, Kai-Uwe Kalies, Karin Römisch

**Affiliations:** 1 Department of Microbiology, Faculty of Natural Sciences and Technology VIII, Saarland University, Campus A1.5, 66123 Saarbrücken, Germany; 2 Center for Bioinformatics, Faculty of Natural Sciences and Technology VIII, Saarland University, 66123 Saarbrücken, Germany; 3 Faculty of Biology, University of Lübeck, Lübeck, Germany

**Keywords:** Protein translocation, Endoplasmic Reticulum, Sec61 channel, ERAD

## Abstract

**Background:**

The Sec61 channel mediates protein translocation across the endoplasmic reticulum (ER) membrane during secretory protein biogenesis, and likely also during export of misfolded proteins for ER-associated degradation (ERAD). The mechanisms of channel opening for the different modes of translocation are not understood so far, but the position of the large ER-lumenal loop 7 of Sec61p suggests a decisive role.

**Results:**

We show here that the Y345H mutation in L7 which causes diabetes in the mouse displays no ER import defects in yeast, but a delay in misfolded protein export. A complete deletion of L7 in Sec61p resulted in viable, cold- and tunicamycin-hypersensitive yeast cells with strong defects in posttranslational protein import of soluble proteins into the ER, and in ERAD of soluble substrates. Membrane protein ERAD was only moderately slower in *sec61∆L7* than in wildtype cells. Although Sec61∆L7 channels were unstable in detergent, co-translational protein integration into the ER membrane, proteasome binding to Sec61∆L7 channels, and formation of hetero-heptameric Sec complexes were not affected.

**Conclusions:**

We conclude that L7 of Sec61p is required for initiation of posttranslational soluble protein import into and misfolded soluble protein export from the ER, suggesting a key role for L7 in transverse gating of the Sec61 channel.

## Background

Protein secretion starts with protein translocation into the endoplasmic reticulum (ER) where secretory proteins mature into a functional three-dimensional conformation before they are packaged into ER-to-Golgi transport vesicles [1]. Proteins that fail to fold in the ER are not allowed to enter these vesicles, and are initially retained in the ER [1]. Most are subsequently exported to the cytosol and degraded by proteasomes, a process called ER-associated degradation (ERAD) [2]. In yeast proteins are imported co-translationally into the ER through a proteinaceous channel formed by the Sec61 complex [3,4]. This heterotrimeric complex consists of the channel-forming Sec61 protein, and two small proteins, Sss1p and Sbh1p, which stabilize the channel and mediate interactions with other protein complexes [3,5,6]. During posttranslational import into the yeast ER the Sec61 channel collaborates with the heterotetrameric Sec63 complex (Sec63p, Sec62p, Sec71p, Sec72p) forming the heptameric Sec complex [3,4]. In yeast transmembrane proteins follow the co-translational pathway, whereas soluble proteins are imported into the ER posttranslationally, and a few primarily ER-resident soluble proteins can use both pathways [4]. Hydrophobicity of the signal sequence determines the mode of translocation, with more hydrophobic sequences leading to co-translational import [7]. The Sec61 channel also plays a role in export of misfolded soluble and transmembrane proteins from the ER as part of a large and likely dynamic complex consisting of an ER-resident ubiquitin ligase and its accessory proteins, the Sec61 channel, Sec63p, but not the other subunits of the Sec63 complex, and the proteasome 19S regulatory particle [1,8].

**Figure 1:**
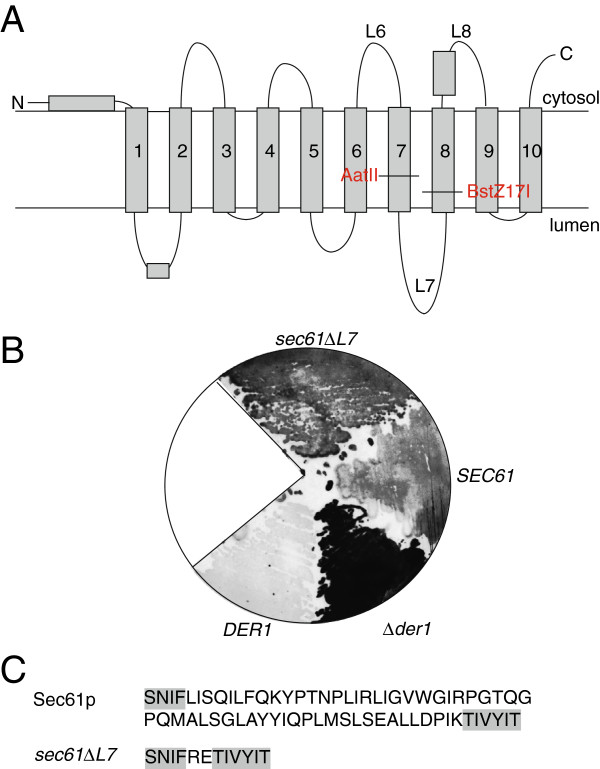
**Identification of a sec61 mutant lacking L7. A)** Topology model of Sec61p including positions of introduced restriction sites. **B)** Yeast (*SEC61*, *sec61∆L7*, *DER1* and *∆der1*) were grown as described in Materials & Methods, transferred to nitrocellulose, lysed, and intracellular CPY* detected by immunoblotting. Note that in the strain used for the *SEC61* mutagenesis there was higher background accumulation of CPY* compared to the *DER1* strain (compare *SEC61* and *DER1*). **C)** Amino acid sequences of L7 and adjacent regions (grey) in wildtype Sec61p and Sec61∆L7p.

Sec61p forms the protein translocation channel which during protein import is almost certainly formed by a single Sec61 complex [3]. Sec61p consists of 10 transmembrane domains (TMDs) with both termini in the cytoplasm, and two large loops, L6 and L8, protruding from the cytoplasmic side of the membrane (Figure 1A) [3]. On the ER lumenal side there is only one large loop, L7 (Figure 1A). Cytoplasmic L6 and L8 are important for binding to the ribosome during cotranslational import into the ER [9]. The structures of the yeast and mammalian Sec61 complexes have so far only been studied by electron microscopy [3]. In the crystal structures of the Sec61 channel orthologue from Archaea, the SecY complex, the 10 transmembrane helices of SecY form a funnel-shaped bundle with a hydrophobic constriction in the center of the channel [3,10]. Cytosolic loops 6 and 8 can be seen clearly protruding from the extracellular face of the membrane [3,10]. On the lumenal side, no protrusions are visible, suggesting that L7 in the crystal was mostly unstructured and hence could not be resolved [10]. L7 almost certainly lies underneath the so-called plug (part of transmembrane domain 2) which closes the hydrophobic constriction through which signal sequences pass from the lumenal side [10]. Thus both the plug and L7 have to move substantially when the Sec61 channel opens transversally for import. Since L7 is the only large extramembrane domain of the channel on the ER-lumenal side it is also likely the point of interaction from which chaperone/misfolded protein complexes trigger channel opening for export of misfolded secretory proteins for degradation in the cytosol.

The importance of L7 for Sec61 channel function is evident from numerous observations: One of the first characterized ER-import defective channel mutants, *sec61-3* (G341E), is located in the center of L7 and causes profound ER import and ERAD defects concomitant with cold- and temperature-sensitivity [11-13]. In an attempt to understand how protein transport across the ER membrane can work at temperatures close to freezing, our laboratory sequenced *SEC61* genes from Arctic and Antarctic fishes and compared them to sequences from temperate fishes [14]. We found that the *SEC61* sequence is extremely highly conserved between fish species, but there were a few amino acid changes primarily in L7 of the polar fishes that we proposed to improve channel function in the cold [14]. Screening mice for genes that cause diabetes Lloyd and colleagues discovered a *sec61* mutant in L7 (Y344H) [15]. The mice had distended ER cisternae in pancreatic beta cells suggesting a defect in ERAD leading to beta-cell death triggered by prolonged induction of the unfolded protein response (UPR) [15]. Y344 was one of the positions in L7 which we had found altered in Arctic fishes [14]. The effects of the Y344H mutation on Sec61 channel function in mammalian cells was investigated by Schäuble et al. [16] who found that it caused an increased calcium leak from the ER through the Sec61 channel which - in contrast to the wildtype channel - could not be switched off by BiP. The authors proposed that in the mutant the Sec61 channel was partially open and suggested that a direct interaction of L7 with BiP was responsible for closure of the wildtype channel [16]. Insertions of HA-tags into L7 at specific positions and replacement with alanine of 4 amino acids which connect the mini-helix in L7 to TMD7 cause a delay in the import of soluble proteins into the ER [17]. Finally, a mutant in L7 (S353C) causes a defect in proteasome-binding to the cytoplasmic surface of the Sec61 channel, suggesting that the conformation of L7 affects the structure of the entire molecule in the membrane [8] (Marie-Luise Kaiser & KR, unpublished).

Because most of Sec61p is embedded in the membrane, mutagenesis of the entire *SEC61* gene predominantly leads to mutations in transmembrane domains [8,13]. In order to be able to mutagenize L7 specifically we introduced restriction sites close to the end of transmembrane domain 7 and the beginning of transmembrane domain 8 (Figure 1A). After L7 mutagenesis we screened the mutants for accumulation of the ERAD substrate CPY*. In these screens we repeatedly isolated *sec61* mutants in which ligation had taken place without an insert. To our surprise, these *sec61∆L7* mutants were viable. Here we describe the characterization of the defects in *sec61∆L7*, and compare them to those of the yeast equivalent of the diabetes-causing mutation in mouse *SEC61*.

## Results

### Yeast expressing *sec61ΔL7* are viable

**Figure 2:**
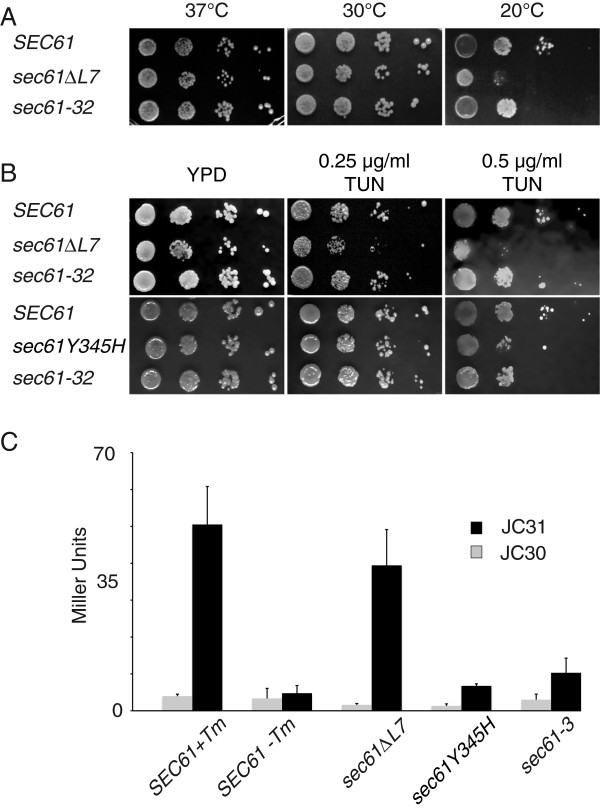
**Viability and growth of *sec61∆L7 *yeast under different stress conditions. A)** Growth of *SEC61*, *sec61∆L7* and *sec61-32* cells was analysed on YPD at 37°C, 30°C (3 d) and at 20°C (7 d). **B)** Tunicamycin sensitivity was examined on plates containing no (left), 0.25 μg/ml (center) or 0.5 μg/ml tunicamycin (right) at 30°C for 6 d. **C)** UPR induction in *SEC61* wildtype and the indicated *sec61* mutant strains was measured as beta-galactosidase activity after transformation of the strains with a UPRE-*LacZ* plasmid (31) or a plasmid encoding *LacZ* alone (30). Wildtype cells treated with 2 μg/ml tunicamycin for 1 h and *sec61-3* cells shifted to 20°C for 1.5 hrs were used as positive controls. Samples were measured in duplicate and the experiment performed 3 times.

In order to be able to investigate functions of L7 of Sec61p, we generated a *sec61* variant with AatII and BstZ17I restriction sites close to the luminal ends of TMDs 7 and 8 (Figure 1A). After mutagenesis, mutant L7 DNA was ligated into the AatII and BstZ17I sites of *sec61*pRS315 and transformed into KRY461 yeast which contained wildtype *SEC61* on a *URA3* plasmid. Transformants were selected on minimal media without leucine, and the wildtype *SEC61* plasmid was counterselected on plates containing 5′- fluoroorotic acid (5′-FOA). We identified L7 mutants of interest by colony-blotting for cells that accumulated the ERAD substrate CPY* intracellularly (Figure 1B) [18]. To our surprise we repeatedly isolated *sec61* mutants in which the AatII and BstZ17I ends of our construct had religated without an insert (*sec61ΔL7*). Compared to a deletion of *DER1*, an ER membrane protein involved specifically in ERAD of soluble secretory proteins, the accumulation of CPY* in *sec61∆L7* was more modest, but still detectable in a screen (Figure 1B, compare *DER1* vs. *∆der1*, and *SEC61* vs. *sec61∆L7*). Upon sequencing we found that in the mutant amino acids 305–371 of Sec61p had been replaced with two amino acids, arginine and glutamate, only, which is equivalent to deletion of the entire L7 and the luminal ends of TMDs 7 and 8 (Figure 1C). TMD7 is part of the lateral gate important for channel opening during secretory protein import into the ER [19,20], and deleting L7 should lead to a decreased flexibility of the channel, thus we expected dramatic translocation defects in *sec61ΔL7* cells*.* We found that nevertheless *sec61ΔL7* cells grew like wildtype cells on plates at 37°C and 30°C; the mutant cells were cold-sensitive at 20°C (Figure 2A). The doubling time for *sec61ΔL7* was increased by 50% (t_gen_ = 2.5 h for *SEC61* and 3.7 h for *sec61ΔL7*). We conclude that L7 of Sec61p, although functionally important, is not essential.

Interference with protein homeostasis in the ER leads to activation of the UPR and hypersensitivity to tunicamycin, which interferes with N-linked glycosylation in the ER and hence with protein folding [21,22]. The Sec61 complex is subject to UPR regulation and translocation-defective *sec61* mutants are frequently UPR-induced and tunicamycin-sensitive [23,24]. When we incubated *sec61ΔL7* yeast on YPD-plates with 0.25 μg/ml or 0.5 μg/ml tunicamycin we found strong tunicamycin sensitivity at 0.5 μg/ml (Figure 2B). The *sec61ΔL7* strain was also sensitive to 0.25 μg/ml tunicamycin in contrast to *sec61-32* cells, the *sec61* mutant with the strongest ERAD defect reported to date (Figure 2B) [13,25]. Tunicamycin-sensitivity of yeast expressing *sec61Y345H* which is homologous to the diabetes-causing *sec61Y344H* in *M. musculus* was similar to *sec61-32* (Figure 2B) [15]. We conclude that *sec61ΔL7* causes strong hypersensitivity to tunicamycin and thus ER stress, indicating a profound disturbance of protein homeostasis in the ER.

To investigate the effect of *sec61* mutants on protein homeostasis in the ER directly, we asked whether *sec61∆L7* or *sec61Y345H* elicited the UPR. We transformed wildtype and mutant strains with a plasmid in which LacZ was expressed under control of a UPR element (pJC31), or without the UPRE as negative control (pJC30), lysed the cells, and analyzed beta-galactosidase activity. As shown in Figure 2C, *sec61∆L7* elicited a very strong UPR, which was almost as strong as the UPR caused by tunicamycin treatment of wildtype cells. UPR induction in *sec61∆L7* was substantially stronger than in *sec61-3* expressing cells, although this mutation had been identified in a screen for UPR-inducing *sec61* mutants (Figure 2C) [24]. UPR induction in *sec61Y345H* cells was modest, but there was a significant difference between cells expressing *UPRE-LacZ* and the control plasmid without the UPRE (Figure 2C). We conclude that L7 of Sec61p is important for maintenance of ER protein homeostasis.

The ER is a repository for Ca^2+^ which is an essential co-factor for chaperones in the ER lumen [26]. In mammalian cells the Sec61 channel is responsible for a Ca^2+^-leak from the ER, and *sec61Y344H* leads to defects in ER Ca^2+^-homeostasis [16,27]. Therefore we investigated whether in yeast *sec61ΔL7* or *sec61Y345H* were defective in Ca^2+^-sealing of the ER by analysing their growth in the presence of the Ca^2+^-chelator EGTA. We detected no effect on growth of either mutant on EGTA, while growth of a strain deleted for the Ca^2+^-pump Pmr1p *(Δpmr1*) was inhibited by 5 mM EGTA (not shown). We conclude that in yeast neither *sec61Y345H* nor *sec61ΔL7* cause gross defects in Ca^2+^-sealing of the ER.

### Deletion of L7 affects soluble protein import into the ER

**Figure 3:**
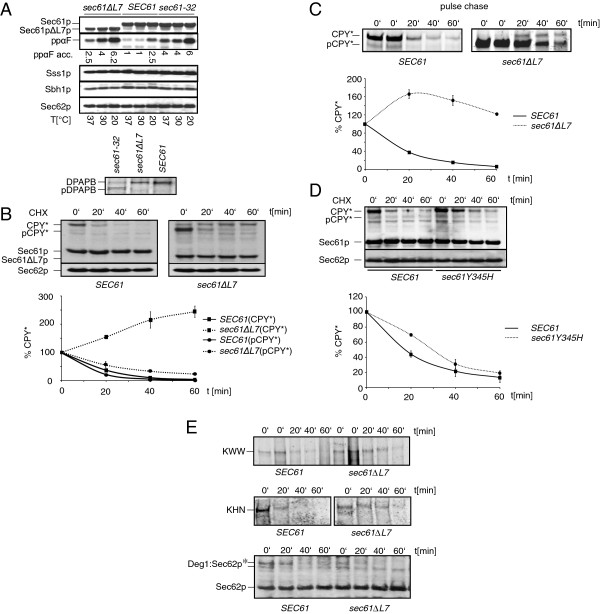
**ER translocation defects in *sec61∆L7 *cells. A)** Yeast were grown o.n. at 30°C to early log-phase and shifted for 3 h to the indicated temperatures. Equal amounts of cells were lysed, proteins resolved on 10% SDS-gels, transferred to nitrocellulose, and detected with specific antibodies. To detect cotranslationally translocated DPAPB, cells were grown to early log phase and pulse labelled for 5 min prior to cell lysis and immunoprecipitation with specific antibodies. **B)** Yeast were grown to an OD_600_ = 1 and translation was inhibited by adding cycloheximide, extracts were prepared by bead-beating, and samples were resolved by SDS-PAGE. Proteins were transferred to nitrocellulose and detected with antibodies against CPY, Sec61p and Sec62p. Quantitation of pCPY* and CPY* are shown in the graph. **C)** Wildtype and *sec61∆L7* yeast expressing CPY* were labelled with [^35^S]-Met/Cys for 5 min and chased for the indicated times, cells were lysed, and pCPY* and CPY* immunoprecipitated. Quantitation of CPY* is shown in the graph. **D)** Cells were grown to OD_600_ = 1 and cycloheximide was added to inhibit translation. Whole cell extracts at 0′, 20′, 40′ and 60′ min were analysed by gel electrophoresis and immunoblotting for Sec61p, CPY* and Sec62p, which served as a loading control. Quantitation of CPY* is shown in the graph. **E)** Wildtype and *sec61∆L7* yeast expressing transmembrane ERAD substrate KWW, or its soluble counterpart KHN were labelled with [^35^S]-Met/Cys for 5 min and chased for the indicated times; cells were lysed, proteins immunoprecipitated and detected by autoradiography. Degradation of the polytopic transmembrane ERAD substrate Deg1:Sec62p was detected in a cycloheximide chase as described in B). Experiments were repeated once (KHN) or twice (KWW, Deg1:Sec62p).

L7 is important for Sec61 channel function in protein transport across the ER membrane (see introduction). We therefore asked whether we were able to detect secretory precursors in lysates of *sec61ΔL7* cells. Soluble prepro alpha factor (ppαF) is posttranslationally transported across the ER-membrane and highly sensitive for defects in translocation. We analysed the accumulation of ppαF in *sec61ΔL7* cells after incubation at 37°C, 30°C and 20°C for 3 h compared to *SEC61*, and *sec61-32* yeast which are cold-sensitive and defective in protein import into the ER [13,25]. Cytosolic accumulation of ppαF was increased in *sec61ΔL7* cells compared to wildtype at all temperatures, and similar to the accumulation in *sec61-32* mutants (Figure 3A). In contrast, cotranslational ER membrane integration of DPAPB was barely affected in *sec61∆L7* cells (Figure 3A, bottom). We next asked whether expression levels of the Sec61p homolog Ssh1p were altered in *sec61∆L7* cells. Ssh1p forms a heterotrimeric complex with Sbh2p and Sss1p which mediates exclusively cotranslational import into the ER [28], and elevation of Ssh1p expression may therefore be able to compensate a cotranslational import defect in *sec61∆L7* cells. We used polyclonal antibodies specific for Ssh1p and determined the ratio of Ssh1p to Sss1p in wildtype and *sec61∆L7* microsomes. We found that in *sec61∆L7* cells, expression of Ssh1p was increased approximately 1.3 fold (not shown). Given that wildtype yeast cells contain 10x less Ssh1 complexes than Sec61 complexes [29] it seems unlikely that this modest elevation in the number of Ssh1 complexes in *sec61∆L7* cells was able to compensate a significant cotranslational import defect in Sec61∆L7 translocons. We conclude that deletion of L7 causes a strong defect in posttranslational import of soluble proteins into the ER.

### Deletion of L7 interferes with soluble misfolded protein export from the ER

The Sec61 channel is a strong candidate for the misfolded protein export channel for ERAD and mutations in *SEC61* result in a delayed export of ERAD substrates to the proteasome in the cytosol [13,25]. Therefore we investigated possible ERAD defects in *sec61∆L7* cells by performing cycloheximide chase and pulse-chase experiments using soluble CPY* as a substrate. CPY* is a substrate for ERAD because of misfolding due to the G255R mutation close to its active site [18]. In a cycloheximide chase monitoring steady state levels of proteins, we found strong accumulation of cytosolic pCPY* in *sec61ΔL7* cells, and only a small amount of CPY* present in the ER-lumen (Figure 3B, right). CPY* degradation was barely detectable in *sec61∆L7* cells resulting in an accumulation of CPY* in the ER-lumen (Figure 3B).

To monitor the fate of newly synthesized CPY* only, proteins were radioactively labelled with [^35^S]-Met/Cys for 5 min, and samples taken every 20 min for up to 1 h. In *sec61ΔL7* cells, posttranslational translocation of newly synthesized pCPY* was dramatically reduced compared to wildtype (Figure 3C, pCPY*, left vs. right). The small amount of translocated CPY* accumulated within the ER initially, but after approximately 30 min, limited ERAD was detectable with slow kinetics compared to wildtype (Figure 3C, right). In wildtype cells CPY* was efficiently imported into the ER and degraded with a t_½_ of less than 20 min (Figure 3C, left). Although it is difficult to differentiate the relative contributions of slow posttranslational import and slow misfolded protein export, the ERAD defect we show here in *sec61ΔL7* cells is the strongest observed for CPY* in any *sec61* mutant characterized so far.

### The diabetes-causing Y345H mutation in L7 delays initiation of ERAD

The mammalian equivalent of the Y345H mutation in Sec61p causes diabetes in the mouse, and dilated ER cisternae in the pancreatic beta cells indicate accumulation of proteins in the ER [15]. We used a cycloheximide chase experiment to determine the effect of the Y345H substitution in yeast Sec61p on CPY* degradation. In three independent cycloheximide chase experiments, we observed a delay in the initiation of degradation of about 20 min (Figure 3D). After 20 min, degradation proceeded with kinetics comparable to the *SEC61* wildtype strain (Figure 3D). Sec61p in *sec61Y345H* cells was stable (Figure 3D). Sec62p served as a loading control and is stable for several hours in cycloheximide chase assays [30]. Our data suggest that similar to the delay in soluble protein import in the L7 mutants generated by Trueman et al. [17] the *sec61Y345H* mutation causes a delay in the initiation of ERAD.

### Deletion of L7 effects on transmembrane protein ERAD

Since we had detected a profound defect in soluble protein transport across the ER membrane in both directions in cells lacking L7 of Sec61p, but none in cotranslational import of transmembrane proteins (Figure 3A, B, C), we decided to also investigate the fate of two transmembrane ERAD substrates in the *sec61∆L7* strain. We first used pulse-chase experiments to determine the half life of the single spanning transmembrane ERAD substrate KWW, and for comparison that of its soluble counterpart KHN [31]. KHN consists of the yeast Kar2p signal peptide fused to the simian virus 5 HA-neuraminidase ectodomain, and is imported into the ER using both the co- and the posttranslational pathway [7]. As expected, it therefore was imported more efficiently into the ER of *sec61∆L7* cells than preproCPY* (compare Figure 3C, E). Nevertheless we observed a dramatic increase in half life for soluble KHN (approximately 15 min in wildtype, 45 min in the mutant; Figure 3E, middle), confirming the ERAD defect for soluble substrates in *sec61∆L7* yeast. In the transmembrane ERAD substrate KWW the simian virus 5 HA-neuraminidase ectodomain is fused to the single membrane-spanning domain of the type I membrane protein Wsc1p [31]. In wildtype cells KWW was degraded with a t_1/2_ of about 30 min (Figure 3E, top left) comparable to its reported t_1/2_ of 35 min [31]. While the t_1/2_ of KWW was slightly increased in *sec61∆L7* cells to approximately 50 min (Figure 3E, top right), the effect of the absence of L7 was modest compared to that on ERAD of soluble substrates.

We next investigated the fate of Deg1:Sec62p, an ERAD substrate with two transmembrane domains and both termini in the cytoplasm, using cycloheximide chase experiments [32]. The cytosolic N-terminus of Deg1:Sec62p contains an N-glycosylation acceptor site which during ERAD is translocated into the ER lumen and modified [32]. Unfortunately, the protein was poorly expressed in our strain background so the determination of its exact half life was problematic, and although we repeated the experiment several times, expression could not be improved. What can be seen on the blot, however, is that the glycosylated form of Deg1:Sec62p, for which ERAD had been already initiated by translocation of the N-terminus into the ER lumen, was degraded with similar kinetics in *SEC61* wildtype and *sec61∆L7* cells (upper band, marked with asterisk, Figure 3E, bottom panel). While in wildtype cells this glycosylated form was dominant (Figure 3E, left, 0 time point), in *sec61∆L7* cells the unglycosylated lower band was more prominent (Figure 3E, right, 0 time point). This lower band was largely stable in *sec61∆L7* cells (Figure 3E, right), demonstrating again that L7 is essential for initiation of ERAD processes that require translocation of a soluble domain across the ER membrane. In contrast entry of TMDs into the lateral gate of the Sec61 channel during ERAD appears to be only moderately dependent on the presence of L7.

### Stability of Sec61∆L7p

Deletion of 66 amino acids resulted in Sec61ΔL7p migrating faster in SDS-gels than wildtype Sec61p (Figure 3A). The amount of Sec61ΔL7p detected by immunoblotting with an N-terminal antibody was only ~70% compared to wildtype and *sec61-32* cells (Figure 3A). The expression levels we observed are similar to those of other *sec61* mutants expressed from plasmids without causing translocation effects [17]. Increasing the expression of Sss1p can suppress the functional defect in Sec61p in *sec61-3* mutants [5]. Therefore we asked whether *sec61ΔL7* cells had elevated their Sss1p levels to maintain viability. We examined the expression levels of Sss1p, Sbh1p and Sec62p, but did not detect any differences between wildtype and *sec61∆L7* mutant cells (Figure 2A). The reduced amount of Sec61ΔL7p in the mutant cells (Figure 3A) may have been due to instability of Sec61p in the absence of L7. We therefore also examined the stability of Sec61ΔL7p in our cycloheximide chase analyses (Figure 3B). Over 1 h, however, Sec61ΔL7p was as stable as the wildtype protein and the Sec62p loading control (Figure 3B).

### The trimeric Sec61 complex is unstable in the absence of L7

**Figure 4:**
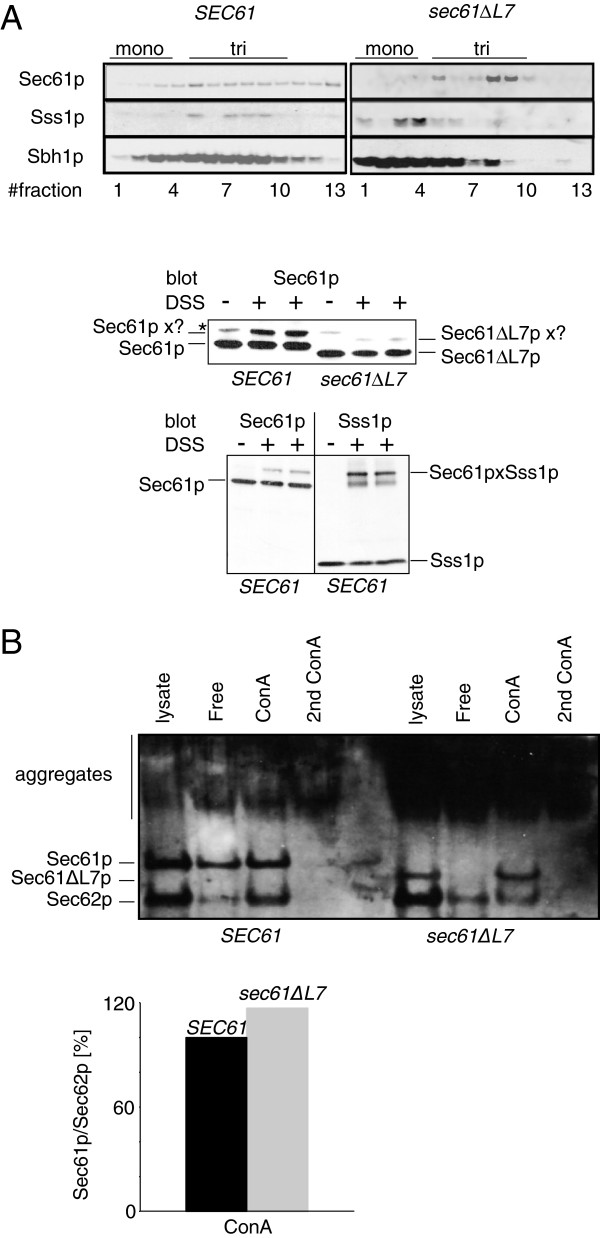
**Stability of Sec61 complex and Sec complex in *sec61∆L7 *membranes. A)** The Sec61 complex is unstable in *sec61∆L7* yeast. Upper: Microsomes from *SEC61* and *sec61∆L7* yeast were solubilised in Triton-X100 and layered onto a 0-15% sucrose gradient. After centrifugation, fractions were collected from the top and proteins resolved by SDS-PAGE. Sec61p, Sss1p and Sbh1p were detected by immunoblotting. Lower: Wildtype and mutant microsomes were treated with 5 mg/ml DSS for 20 min at 20°C. After quenching the crosslinked proteins were resolved by SDS-PAGE and Sec61p- and Sec61∆L7p-containing crosslinks were detected by immunoblotting with anti-Sec61p antibodies or anti-Sss1p antibodies. The asterisk marks a background band that is independent of crosslinking and migrates slightly slower than the Sec61pxSss1p band. **B)** Microsomes were solubilized in digitonin and centrifuged at high speed to remove ribosome-bound Sec61 complex. From the cleared lysate, the heptameric Sec complec was precipitated with ConcanavalinA-Sepharose, and Sec61p and Sec62 in supernatant and precipitates detected by immunoblotting. Note that the gel is overexposed to show the substantial fraction of Sec61∆L7p found in SDS-resistant aggregates at the top of the gel. Ratios of Sec61p to Sec62p in wildtype and mutant Sec complexes are shown in the graph.

We next asked whether instability of any of the protein complexes formed with Sec61p was the explanation for the protein translocation defects observed in *sec61∆L7* cells. The trimeric Sec61 complex, which consists of Sec61p, Sss1p and Sbh1p, is stable in Triton-X100, in contrast to the heptameric Sec complex [33]. We solubilized microsomes derived from wildtype and *sec61∆L7* cells in Triton-X100 and analysed Sec61 complex integrity by sedimentation in a 0-15% sucrose gradient. After centrifugation, fractions were taken from the top, proteins separated by SDS-PAGE, and Sss1p, Sbh1p and Sec61p detected by immunoblotting. The stable trimeric Sec61 complex was located in fractions 5–10 where Sec61p, Sss1p and Sbh1p were detectable in microsomal lysates from *SEC61* wildtype yeast (Figure 4A, left). In lysates from *sec61ΔL7* membranes, substantial fractions of Sbh1p and Sss1p were found in fractions 1–4 which represent the monomeric states of Sss1p and Sbh1p (Figure 4A, right). This suggests that Sec61ΔL7p fails to bind Sbh1p and Sss1p appropriately, and that this leads to an instability of the trimeric Sec61 complex. The effect was most striking for Sss1p, which in the *sec61∆L7* mutant was found almost exclusively in the monomeric fraction (Figure 4A, right). The distribution of Sec61ΔL7p in the gradient also changed compared to wildtype Sec61p: it was found concentrated in fractions 8 and 9 where no Sss1p and little Sbh1p was present (Figure 4A, right). Surprisingly, in contrast to the small subunits, no Sec61∆L7p was found in the monomeric fractions on the top of the gradient (Figure 4A, right, fractions 1–4).

To confirm the altered interaction of Sec61∆L7p with the small subunits of the Sec61 complex we performed a chemical crosslinking experiment. In mammalian microsomes, chemical crosslinking with sulfhydryl-reactive bifunctional bis-maleimidohexane (BMH) results in a prominent band consisting of the Sec61p homologue Sec61α and the Sbh1p homologue Sec61β [34]. This crosslink is sensitive to structural changes in the translocon and disappears upon treatment of the membranes with EDTA, and after stripping off ribosomes with puromycin and high salt [34]. We treated wildtype or *sec61∆L7* mutant yeast microsomes with the amine-reactive homobifunctional crosslinker disuccinimidyl suberate (DSS) which has approximately the same linker length as BMH but more potential target amino acids in Sec61p, Sss1p and Sbh1p. Crosslinking of wildtype membranes resulted in a single prominent crosslinked band which was about 10 kD larger than Sec61p (Figure 4A, center panel). Immunoblotting on the crosslinked material with antibodies against Sbh1p and Sss1p revealed that this band contained primarily Sec61p/Sss1p heterodimers (Figure 4A, lower panel), but a very modest amount of Sec61p/Sbh1p heterodimers was also detected (not shown). In *sec61∆L7* microsomes, the crosslink was at least 5-fold weaker compared to wildtype membranes confirming changes in the interactions of Sec61∆L7p with Sss1p (Figure 4A, center panel, compare + DSS for *SEC61* and *sec61∆L7*). We conclude that L7 of Sec61p is essential for hetero-oligomeric stability of the Sec61 complex, and thus for stability of the Sec61 channel.

### Loss of L7 does not affect Sec61 complex interaction with the Sec63 complex

The heptameric Sec complex consists of the trimeric Sec61 complex associated with the Sec63 complex comprising Sec62p, Sec63p, Sec71p and Sec72p. Sec71p is the only glycosylated Sec complex subunit; association of the Sec61 complex with the Sec63 complex can therefore be demonstrated by co-precipitation of Sec61p with the lectin ConcanavalinA [13]. The heptameric Sec complex is stable in digitonin. To ask whether L7 deletion in Sec61p had any effect on formation of the Sec complex, we solubilized wildtype and *sec61∆L7* microsomes in digitonin and removed ribosome-bound Sec61 complexes by ultracentrifugation (digitonin lysate). From the lysate, we precipitated the heptameric Sec complex using ConcanavalinA-Sepharose and analysed both the amount of free Sec61 complex in the supernatant and the amount of ConcanavalinA-associated Sec61 complex by Western Blotting (Figure 4B). Saturation of the precipitation was controlled by a second ConcanavalinA precipitation from the supernatant (control). In lysates from *SEC61* wildtype membranes, the amount of Sec61p in the free-fraction was 25-30%, and the remainder was found with the heptameric Sec complex in the ConcanavalinA-bound fraction (Figure 4B, left). The amount of digitonin-solubilized Sec61ΔL7p was substantially lower than that of the wildtype protein, and its distribution was also different: almost all detectable Sec61ΔL7p was found in the ConcanavalinA-bound fraction, and little if any in the free fraction (Figure 4B, right). Inspection of the upper part of the gel showed that Sec61∆L7p forms SDS-resistant aggregates in digitonin, in contrast to wildtype Sec61p (Figure 3B, bar). The ratios of wildtype or mutant Sec61p to Sec62p, however, were similar in the ConcanavalinA-bound fractions (Figure 4B) suggesting no dramatic effects of the L7 deletion on heptameric Sec complex formation.

### Loss of L7 does not interfere with binding of proteasomes to the Sec61 complex

**Figure 5:**
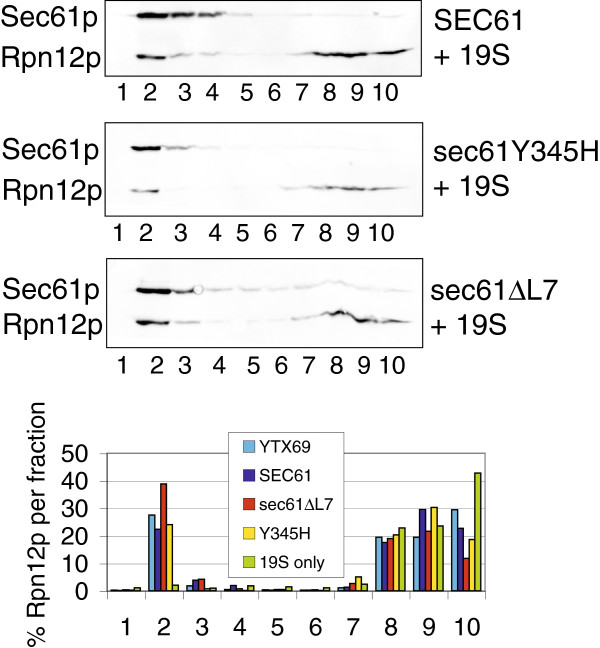
**Proteasome 19S particle binding to wildtype, *sec61Y345H *and *sec61∆L7 *proteoliposomes.** Microsomes from wildtype or *sec61* mutant yeast were prepared, stripped of ribosomes, solubilized, and total protein reconstituted into proteoliposomes. Membranes were incubated with purified 19S particles in the presence of 5 mM ATP, and samples analyzed by flotation in 1.8 M sucrose for 1 h at 200,000 g. Fractions were collected from the top and analyzed by SDS-PAGE and immunoblotting for Sec61p and the 19S subunit Rpn12p. Rpn12p in each fraction was quantified. Note that 19S particles in the absence of membranes (green) remain at the bottom of the gradient. YTX69 is the standard wildtype yeast strain used in the lab for proteasome and ribosome binding experiments; *SEC61*, *sec61Y345H*, and *sec61∆L7* were all in the KRY461 background.

Numerous mutations in *SEC61* affect export of misfolded proteins from the ER to the cytosol for degradation by proteasomes [25,13,24; this work]. In addition, proteasomes can bind directly to the Sec61 channel, and a specific mutation in L7 affects proteasome binding [8,35] (Marie-Luise Kaiser & KR, unpublished). We therefore asked whether the *Y345H* mutation or deletion of L7 had any effects on the interaction of the Sec61 channel with proteasomes. We had observed previously that solubilization of yeast membranes and reconstitution of total protein into proteoliposomes improved proteasome binding to the membranes [35]. We therefore prepared proteoliposomes from wildtype, *sec61Y345H* and *sec61∆L7* puromycin/high salt-treated microsomes and performed binding experiments with purified yeast 19S proteasome particles as described [35]. As shown in Figure 5, we found no differences in proteasome binding between wildtype (blue) and *sec61Y345H* (yellow) proteoliposomes. Binding of 19S particles to *sec61∆L7* proteoliposomes consistently was slightly higher than to wildtype *SEC61* proteoliposomes (Figure 5, red vs. blue). We conclude that the ERAD defects observed in *sec61Y345H* and *sec61∆L7* yeast are not due to defects in proteasome interaction with the Sec61 channels in the ER membrane.

## Discussion

In this paper we have characterized a new *sec61* mutant, *sec61∆L7*, which lacks the functionally important ER-lumenal loop 7 and the adjacent ends of TMDs 7 and 8 (Figure 1A, C). The deletion shortens TMD7 of Sec61p to 14 amino acids which on its own is too short to span a bilayer [36]. In the context of a polytopic membrane protein, however, the hydrophobic mismatch of an individual short TMD during membrane integration can be compensated by the surrounding TMDs which stabilize the short segment in the membrane [36]. Our data suggest that the topology of Sec61∆L7p was unaltered as cells expressing *sec61∆L7* as sole copy of *SEC61* were alive and growing (Figure 2). Sec61∆L7p was expressed only to about 70% of wildtype protein levels (Figure 3A), and while the protein was stable in a cycloheximide chase (Figure 3B) our data cannot exclude a slight defect early in Sec61∆L7p biogenesis. In cells expressing *SEC61* from a *GAL* promoter, however, protein levels need to be reduced well below 50% before translocation defects occur, and heterozygous diploids with only one functional copy of *SEC61* do not have ER translocation defects (Louise Hutt & KR, unpublished). It therefore seems unlikely that the expression level of the mutant protein per se was the cause for the translocation defects observed.

The *sec61∆L7* mutant was more sensitive to cold and tunicamycin than *sec61-32* cells, and displayed a stronger UPR induction suggesting a more severe disturbance of ER translocation and ER protein homeostasis than in the *sec61* allele with the strongest ERAD defect identified previously (Figure 2) [13,25]. Mutant *sec61∆L7* cells strongly accumulated soluble posttranslationally translocated preproalpha factor in the cytosol (Figure 3A), and displayed a profound import defect for soluble posttranslationally translocated pCPY* in both cycloheximide chase and pulse-chase experiments (Figure 3B, C). Association of the Sec61∆L7 complex with the Sec63 complex was normal (Figure 4B), however, so the defect in posttranslational import must be due to a functional defect in the heptameric complex.

**Figure 6:**
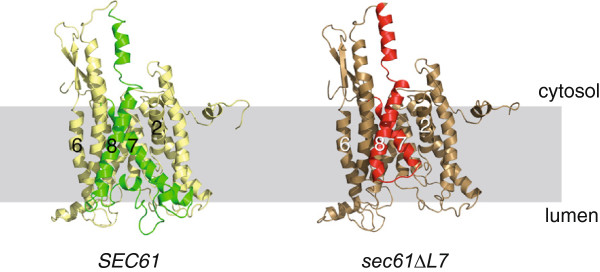
**Homology models of wildtype Sec61p and Sec61∆L7p in the ER membrane.** Side view of homology model of wildtype Sec61p (left) and Sec61∆L7p mutant (right). TM7, TM8 and their connecting loop (Loop 7) are highlighted in green (wildtype) and red (∆L7). Parts of the protein that remain unchanged are shown in shades of yellow. The membrane is indicated in light gray.

Although the solubilized Sec61∆L7 complex was unstable (Figure 4A), cotranslational membrane integration of DPAPB was barely affected (Figure 3A). Modelling of the Sec61∆L7 mutant protein suggests that structural changes are limited largely to the ER-lumenal face of the Sec61 channel, and that the cytoplasmic surface of the channel remains similar to wildtype (Figure 6). Combined with our experimental data this indicates that ribosome binding to Sec61∆L7 channels can proceed normally and ribosome binding likely stabilizes trimeric Sec61∆L7 channels such that subsequent channel opening can proceed in the absence of the lumenal end of the lateral gate and L7.

Ribosomes and proteasomes bind to different regions of the cytoplasmic face of the Sec61 channel, but the largely unaltered cytoplasmic surface of the Sec61∆L7 channel likely also explains why proteasome binding was not reduced [8] (Figures 6 and 5). We were suprised by this observation because we had found previously that a point mutation in L7, S353C, reduces proteasome affinity for the Sec61 channel [8] (Marie-Luise Kaiser & KR, unpublished). It therefore appears that when it is present the conformation of L7 is important for proteasome interaction with the channel, and that conformation of L7 can be transmitted through the transmembrane helices to the cytoplasmic face of the channel. Our data regarding proteasome binding to Sec61∆L7 channels suggest that the defect in soluble misfolded protein export in *sec61∆L7* cells shown in Figure 3 is not due to reduced proteasome binding.

The relative contributions of slow import and slow export to the profound ERAD defect in *sec61∆L7* cells are difficult to differentiate for posttranslationally imported substrates (Figure 3B, C). We observed progressive accumulation of soluble CPY* in the ER over time (Figure 3B) which suggests that export may be even slower than import, possibly because there is a direct competition of the two processes for common factors (Sec61p, BiP, Sec63p) [8,37]. This phenotype is similar to the result of overexpression of CPY* where increasing the load on the ER-to-cytosol transport pathway causes cytosolic accumulation of secretory precursors which could be alleviated by increasing the expression of *SEC61 *[38]. Co-translational membrane protein integration was barely affected in *sec61∆L7* (Figure 3A, lower panel). The strong defects in soluble protein import and export through the Sec61∆L7 channel indicate that in the absence of L7 the channel can no longer open properly in the transverse direction. While integration of membrane proteins via lateral channel opening towards the lipid bilayer is still possible, and re-entry of simple transmembrane ERAD substrates is only moderately delayed, transport of soluble proteins through the channel in either direction is strongly impeded, and the general slowdown in transport might lead to competition of biosynthetic soluble protein import and misfolded soluble protein export for ERAD.

Import of KHN mediated by the BiP signal peptide which can use both posttranslational and cotranslational import pathways was barely affected in *sec61∆L7* cells (Figure 3E, middle panel; [7,31]). This suggests that BiP likewise will be translocated efficiently into the ER in *sec61∆L7* cells, and when we blotted on wildtype and mutant extracts we did not detect cytosolic BiP precursor in *sec61∆L7* cells (not shown). ERAD of KHN, however, was strongly defective in the *sec61∆L7* mutant (Figure 3E, middle panel) in contrast to ERAD of its membrane-anchored counterpart KWW whose half life increased only moderately (Figure 3E, top panel). Since KHN and KWW have been shown by Vashist and Ng [31] to have identical chaperone requirements for ERAD, this experiment demonstrates that - rather than affecting indirectly the chaperone composition in the ER lumen - *sec61∆L7* has a direct negative effect on export from the ER of soluble substrates only.

The *sec61Y345H* mutant had no growth defect at any temperature (not shown), and a tunicamycin sensitivity comparable to *sec61-32* (Figure 2B) and *sec61-3 *[39]. It was fully functional in protein import into the ER (not shown, and Figure 3D), and had only a modest defect in ERAD of CPY* (Figure 3D) [39]. That *sec61Y345H* causes an ERAD defect in the absence of a secretory protein biogenesis defect confirms the direct role of Sec61p in ERAD. Strikingly, *sec61Y345H* caused a delay in initiation of ERAD rather than absolute slower kinetics of export for degradation (Figure 3D) [39] suggesting that this position in L7 might play a role in the initiation of Sec61 channel opening from the lumenal side for export of ERAD substrates. One would expect a mild phenotype in order for mice to survive this mutation in an essential gene [15]. Delayed ER export in pancreatic beta cells which have a high secretory protein load would result in gradual ER accumulation of misfolded proteins, followed by cell death, and the development of diabetes as a primary phenotype [15]. The delay in the initiation of ERAD in *sec61Y345H* yeast is reminiscent of the delay in protein import observed by Trueman et al. [17] in L7 mutants that disrupt the interaction of L7 with TMD7. Taken together, our data suggest that L7 conformation is crucial for Sec61 channel gating for both import and ERAD of soluble proteins.

Modelling of the Sec61∆L7 protein suggests that the ‘plug’ formed by transmembrane helix 2a remains in place, but the lateral gate formed by interaction of transmembrane helix 2b with transmembrane helix 7 is partially open, as helix 2b is shifted significantly towards the cytoplasmic surface of the membrane (Figure 6). This shift is likely the consequence of the missing lumenal end of TMD7 which can no longer interact with helix 2b and hold it in place. The deletion in Sec61∆L7p begins 2 amino acids C-terminal of N302 which is the most C-terminal residue of the gating motif responsible for setting the hydrophobicity threshold for entry of signal sequences into the Sec61 channel [20]. Destabilizing the gating motif by replacing N302 with more polar amino acids causes promiscuous insertion of even marginally hydrophobic signal peptides into the gate [20]. In Sec∆L7p N302 is under strain because it is now close to the end of truncated TMD7 which is connected to TMD8 by only 2 amino acids (Figure 6). This will weaken the hydrogen bonds to N302 partners in the gating motif which likely explains the partial opening of the gate (Figure 6). While the destabilization of the lateral gate in the Sec61∆L7 channel is similar to that of the N302-to-polar mutants, in contrast to Trueman et al. [20] we do not see enhanced import of soluble proteins by the Sec61∆L7 channel, but rather an almost complete block of transport of soluble proteins into and out of the ER (Figure 3). Both we and Trueman et al. [20] observed much milder effects or none on the integration of transmembrane proteins into the ER in our respective mutants (Figure 3A) [20]. Trueman et al. did not investigate the effects of their L7 and gating motif mutants on ERAD [17,20].

## Conclusions

We have shown here that a Sec61p mutant lacking ER-lumenal loop 7 displays severe ERAD defects for soluble substrates (Figure 3B, C, E). In contrast, ERAD of single-spanning KWW was only moderately slower than in wildtype yeast (Figure 3E). For soluble misfolded protein export to the cytosol through the Sec61 channel L7 is the only possible starting point, because it is the only large extramembrane domain of the channel in the ER lumen. If L7 is missing, chaperone/export substrate complexes have no contact point from which to open the lateral gate, and exit from the ER is compromised; transmembrane proteins, however, can still enter laterally into the gate using their hydrophobic TMDs. Collectively, our data suggest that lateral gate opening of the Sec61 channel for entry or exit can proceed independently of L7, whereas transverse gating for soluble protein transport in either direction requires the presence of L7.

## Methods

### Yeast strains & growth conditions

Two restriction sites surrounding L7 (4th luminal loop) were introduced within the *SEC61* ORF by site-directed mutagenesis using the Strategene kit. After restriction with AatII and BstZ17I, self-ligation of the *ORF* resulted in *sec61ΔL7*pRS315. In *sec61ΔL7*pRS315, amino acids 305–371 of wildtype Sec61p had been replaced by two amino acids only: arginine, glutamate (RE). Point mutants *sec61Y345H* and *sec61-32* were established by site-directed mutagenesis in bacterial pUC19-vector and cloned into yeast plasmid pRS315, resulting in *sec61Y345H*pRS315 and *sec61-32*pRS315. The plasmids were transformed into KRY461 (*SEC61::HIS3 ade2-1 leu2-3, 112 trp1-1 prc1-1 his3-11, 15 ura3-1* [pGAL-*SEC61*-*URA3*]), selected on minimal medium without leucine, then on 5′-FOA in minimal medium without leucine at 30°C for 4 d, and used for assays described below. Solid media: Yeast were grown in YPD to an OD_600_ of 0.8-1.5 and counted in a Neubauer-Chamber. Cells (10^4^-10^1^) were dropped on YPD-plates without or with 0.25 μg/ml Tunicamycin, 0.5 μg/ml Tunicamycin or 5 mM EGTA. Plates were incubated for 3 d (37°C, 30°C) or 7 d (17°C, 20°C, EGTA, tunicamycin) and growth was examined. Liquid media: YPD was inoculated to an OD_600_ = 0.005 or 5 x 10^4^ cells/ml and growth was monitored at 2 h intervals by counting in a Neubauer chamber or by photometric measuring at 600 nm. YTX69 (*MATa/α his3-11,15/his3-11,15 leu2-3,112/leu2-3/112 trp1-1/trp1-1 ura3-1/ura3-1 ade2-1/ade2-1 can1-100/can1-100*).

### UPR assay

*SEC61* wildtype and mutant cells were transformed with pJC30 (LacZ) or pJC31 (UPRE-LacZ), and beta-galactosidase activity was assayed after growth overnight in SC without Trp to early log phase. Cells (2 ml) were harvested by centrifugation and resuspended in 1 ml Z-buffer (60 mM Na_2_HPO_4_, 40 mM NaH_2_PO_4_, 10 mM KCl, 1 mM MgSO_4_, 0.27% mercaptoethanol) and yeast were lysed with 100 μl chloroform, 50 μl 0.1% SDS and vortexing for 10 sec. Suspension was preincubated for 5 min at 28°C and 200 μl ONPG (4 mg/ml) were added. After 30 min, the reaction was stopped by adding 700 μl Na_2_CO_3_, the absorbance at 420 nm was determined and Miller units were calculated. *SEC61* yeast were treated with tunicamycin (2 μg/ml) for 1 h and *sec61-3* cells were incubated at 20°C for 1.5 h.

### Colony blot

Yeast were grown on minimal medium (2% galactose, 0.67% YNB, 2% agar, 0.2% amino acid mix without Leu) at 30°C for 2 d and transferred to nitrocellulose. Nitrocellulose was incubated for 2 d upside down on minimal medium with 1% potassium acetate to increase CPY* expression, and 10 h on minimal medium with 4 μg/ml cycloheximide. Cells were lysed in lysis buffer (0.1% SDS, 0.2 M sodium hydroxide, 0.5% β-mercaptoethanol) and carefully washed with TBS-T. CPY* levels were detected by immunoblotting with a specific polyclonal antibody against CPY (our lab).

### Secretory precursor accumulation at different temperatures

Yeast cells were grown overnight at 30°C to an OD_600_ = 1 and incubated for 3 h at 37°C, 30°C or 20°C. Equal amounts of cells were lysed in 100 μl SDS-sample buffer (with 200 mM DTT) with glass beads in a bead beater for 2× 1 min. Extracts were heated to 65°C for 10 min and equivalents of 0.3 OD loaded for every lane onto 10% SDS-PAGE gels. Proteins were separated in MOPS-buffer, transferred to nitrocellulose and bands were detected by immunoblotting with specific primary antibodies (Sbh1p, Sec61p and ppaF: Römisch lab (1:2000); Sec62p, Sss1p: Schekman lab (1:4000)). Primary antibodies were detected with anti-rabbit-HRP antibodies (1:10000; Rockland) and visualized with ECL (Thermo-Fisher). Pho8p and DPAPB were detected by immunoprecipitation from 1 OD cells labelled for 5 min with [^35^S]Met/Cys-Mix (Promega) as below.

### Pulse-chase

Yeast were grown overnight at 30°C in minimal medium without leucine (0.2% CAA, 5% Glc, 0,67% YNB without amino acids/with NH_4_SO_4_) to OD_600_ = 1. Cells were washed with labelling medium (5% Glc, 0,67% YNB without amino acids and ammonium, supplemented with amino acids without Cys and Met) and concentrated to 4 OD/ml. For each time point, 250 μl of the suspension were starved for 20 min at 30°C in labelling medium and pulsed for 5 min with 55 μCi [^35^S]Met/Cys-Mix (Promega). For chase experiments, to each sample an equivalent volume of 2x chase-mix (10 mg Cys, 30 mg Met, 200 mM NH_4_SO_4_) in labelling medium was added and stopped by adding 500 μl ice-cold Tris-azide (20 mM Tris, pH 7.5, 20 mM sodium azid). The cells were washed with Tris-azide, resuspension solution (100 mM Tris, pH 9.4, 10 mM DTT, 20 mM sodium azid) and resuspended in 150 μl lysis buffer (20 mM Tris, pH 7,5, 2% SDS, 1 mM DTT, 1 mM PMSF) and half a volume of acid-washed glassbeads (Sigma). Samples were lysed in a bead beater and proteins denaturated for 10 min at 95°C. Proteins were immunoprecipitated, precipitates denatured for 5 min at 95°C in sample buffer, and resolved on 10% SDS-PAGE (NuPage) in MOPS-buffer and bands detected by autoradiography.

### Cycloheximide chase

Yeast were grown overnight to an OD_600_ = 1 and treated with 200 μg/ml cycloheximide (t = 0). An equal amount of cells (5 OD) were removed every 20 min for 60 min and washed with ice-cold Tris-azide to kill the cells. Yeast were lysed with glass beads in a bead beater for 2× 1 min in SDS-sample buffer and lysates heated to 65°C for 10 min. After gel electrophoresis on 10% SDS-PAGE in MOPS-buffer CPY*-levels were detected by immunoblotting with CPY-antibodies (Römisch lab; 1:2000) and continued as described above.

### Stability of the trimeric Sec61 complex in sucrose gradient centrifugation

Microsomes were prepared as described in [25]. A sucrose gradient was prepared from 1 ml 15%, 10%, 5% and 0% [w/v] sucrose in 50 mM HEPES-KOH, pH 7.5, 500 mM potassium acetate, 1 mM EDTA, 0.1% Triton X-100, 0.05% β-mercaptoethanol, 1 mM PMSF, and 1× protease inhibitor cocktail and allowed to form for 6 h at room temperature. Microsomes (50 eq) were sedimented (10000 g, 4°C, 1 min) and the pellet resuspended in solubilization buffer (100 μl of 50 mM HEPES-KOH, pH 7.5, 500 mM potassium acetate, 1% Triton X-100, 10 mM EDTA, 0.05% [v/v] β-mercaptoethanol, 1 mM PMSF, and 5x protease inhibitor cocktail), for 15 s agitated on a Vortex Mixer and incubated for 15 min on ice [33]. The solubilized microsomes were layered on a 0-15% sucrose gradient and centrifuged in an ultracentrifuge (SW55Ti, 200,000 g, 4°C, 16 h). After centrifugation, 13 fractions of 310 μl each were collected from the top, precipitated with TCA, resuspended in 40 μl SDS-sample buffer, heated to 65°C for 10 min and protein resolved by SDS-PAGE. Proteins were transferred to nitrocellulose and incubated with the indicated antibodies as described above.

### Crosslinking of the Sec61 complex in intact microsomes

Microsomes (5 μl of OD_280_ = 30) were crosslinked in 50 μl B88, pH 7.9, by addition of 6 μl freshly made 5 mg/ml DSS (Thermo Fischer) in dry DMSO. After 20 min at 20°C crosslinking was quenched by addition of 7.5 μl 8.4 M ammonium acetate. Proteins were denatured in SDS sample buffer at 65°C, separated by SDS-PAGE and Sec61p, Sbh1p and Sss1p detected by immunoblotting with specific polyclonal antisera.

### Isolation of the heptameric Sec complex

Microsomes were prepared as described in [25]. Microsomes (50 eq) were sedimented (10000 g, 4°C, 1 min) and the pellet was resuspended in 100 μl solubilization buffer (50 mM HEPES-KOH pH 7.4; 400 mM potassium acetate; 5 mM magnesium acetat; 10% (w/v) Glycerol; 0.05% (v/v) β-Mercaptoethanol, 1x PI). Solubilization buffer (400 μl) with 3.75% [w/v] digitonin was added, and membranes solubilized for 30 min on ice. Insoluble debris were removed by centrifugation (10000 g, 4°C, 10 min), the supernatant collected and the pellet treated again with 300 μl solubilization buffer with 3.75% [w/v] digitonin and centrifuged again. The resulting supernatant was united with the first supernatant (~800 μl) and centrifuged in an ultracentrifuge (TLA100.3, 70 min, 200000 g, 4°C) to remove the ribosome-associated heterotrimeric Sec61-complex. The supernatant was subsequently referred as “digitonin extract”. Solubilization buffer (450 μl) was added to 150 μl digitonin extract and heptameric Sec-complex containing the Sec71p glycoprotein precipitated with ConA-Sepharose (1 h, 4°C). To control for the saturation of the ConA-precipitation, the supernatant was centrifuged for 10 min, 4°C and 10000 g, and precipitated again with 100 μl ConA-Sepharose (1 h, 4°C). The supernatant was collected (referred to as “free fraction”). Both, ConA-precipitates were centrifuged (2500 g, 2 min) and washed with equilibration buffer (1% [w/v] digitonin; 50 mM HEPES-KOH pH 7.4, 10% [w/v] Glycerol, 0.05% [v/v] β-mercaptoethanol, 1x PI). This step was repeated 2×. The ConA-beads and the TCA-precipitated extracts were resuspended in 40 μl SDS-sample buffer with DTT, heated to 65°C for 10 min and resolved in SDS-PAGE as described above.

### Proteasome binding

Proteasomes were isolated and proteasome binding experiments to proteoliposomes performed as in Kalies et al. [35].

### Modelling of Sec61∆L7p

We homology modeled *S. cerevisiae* Sec61p and Sec61∆L7p using the software MODELLER 9.10 [40]. In order to obtain better homology models we used the multi template homology modeling approach with default parameters. We identified the templates considering both sequence similarity and resolution of the crystal structures. The putative templates can be classified into two groups: The first group consists of prokaryotic crystal structures with relatively low sequence similarity whereas the second group comprises the relatively more similar eukaryotic structures which were obtained by fitting homology models to EM structures. We decided to combine the power of those two groups. The used templates are as follows:

2WWB (Chain A): *Canis lupus familiaris*, Sec61 alpha (Homology model was fitted to Cryo-EM image); 2WW9 (Chain A): *S. cerevisiae*, Sec61p (Homology model was fitted to Cryo-EM image); 3MP7 (Chain A): *Pyrococcus furiosus*, SecY; 1RH5 (Chain A): *Methanococcus jannaschii*, SecY. We chose the best models according to both DOPE and molpdf evaluation scores. We placed Sss1p into our model using the cryo-EM structure of the yeast Sec61 complex with the pdb code 2WW9 [41]. First we superimposed the Sec61p homologue (2WW9, chain A) and the best homology models of both the wildtype and the ∆L7 mutant. Afterwards we copied Sss1p (2WW9, chain B) into our model. The position of the membrane was predicted using the method of Lomize et al. [42]. The end points of the membrane correspond to locations of lipid carbonyl groups.

## Competing interests

The authors declare no competing interests related to this article.

## Authors’ contributions

TT and KR designed the experiments, interpreted the data, and wrote the paper. TT performed the experiments characterizing the *sec61∆L7* mutant, FP performed the crosslinking experiment, SA and KK performed the proteasome binding experiments, and OU and VH modelled the mutant protein. All authors read and approved the final manuscript.
